# Assessment of three broadleaf weed species classification in rice field using UAV hyperspectral imaging and machine learning

**DOI:** 10.3389/fpls.2025.1662972

**Published:** 2025-12-18

**Authors:** Nursyazyla Sulaiman, Nik Norasma Che’Ya, Abdul Shukor Juraimi, Nisfariza Mohd Noor, Rhushalshafira Rosle, Muhammad Huzaifah Mohd Roslim

**Affiliations:** 1 Department of Agriculture Technology, Faculty of Agriculture, Universiti Putra Malaysia, Serdang, Selangor, Malaysia; 2 Department of Crop Science, Faculty of Agriculture, Universiti Putra Malaysia, Serdang, Selangor, Malaysia; 3 Department of Geography, Faculty of Arts and Social Sciences, University of Malaya, Kuala Lumpur, Malaysia; 4 Department of Crop Science, Faculty of Agricultural Science and Forestry, Universiti Putra Malaysia, Bintulu, Sarawak, Malaysia

**Keywords:** hyperspectral, remote sensing, machine learning, rice crop, weed

## Abstract

Broadleaf weed (BLW) infestation is a major challenge in rice cultivation, particularly during the early vegetative stages when competition for resources is most critical. This study aims to enhance early-stage detection and classification of three prevalent BLW species—*Monochoria vaginalis* (MV)*, Limnocharis flava* (LF), and *Sphenoclea zeylanica* (SZ)—in rice fields using unmanned aerial vehicle (UAV)-based hyperspectral imaging integrated with machine learning techniques. The research was conducted in a 1-hectare rice plot (Block L5A, Plot 121) near Pusat Benih Padi Felcra Sdn Bhd, Perak, Malaysia, a site characterized by high weed density. Hyperspectral data were acquired using a DJI Matrice 600 UAV equipped with a Resonon Pika L hyperspectral camera flown at 40 meters altitude. ENVI Classic 5.3 software was used to perform supervised classification based on selected regions of interest (ROIs) for training. Three classification algorithms—Support Vector Machine (SVM), Minimum Distance (MD), and Parallelepiped (PP)—were compared at 15, 25, and 30 days after sowing (DAS). Among them, SVM consistently achieved the highest classification accuracy, exceeding 99% for all weed species across all growth stages, with minimal omission and commission errors. Vegetation cover analysis showed an increasing trend in BLW expansion over time, while rice cover fluctuated and soil cover declined, indicating the competitive dominance of weeds. The findings underscore the effectiveness of UAV hyperspectral imaging combined with machine learning—especially SVM—as a scalable, accurate, and efficient approach for early weed detection. This methodology can support precision agriculture by enabling timely and targeted weed management strategies, ultimately improving rice yield and sustainability.

## 1 Introduction

Weeds are biological components of agricultural ecosystems and are generally defined as unwanted plants that interfere with human activities or are considered visually undesirable. They represent a significant cause of crop yield loss for farmers (Haq et al., 2024). In rice cultivation, weeds pose the greatest threat as they compete with rice plants for essential resources such as nutrients, light, water, and space (El-Ghandor, 2024). Globally, there are around 30,000 weed species, of which approximately 18,000 are known to cause significant crop losses (Sai et al., 2022). The magnitude of these losses is influenced by multiple factors, including the severity and timing of weed infestation as well as the specific weed species present, since all plants compete for essential resources such as nutrients and water required for growth and establishment (Kanatas et al., 2021; Colbach et al., 2023). Weed infestations can typically reduce rice yields by 10% to 20%, and in cases of severe infestation, the entire crop may be lost (Gao et al., 2022). The degree of damage caused by weeds in rice fields is influenced by factors such as weed species, their density, and the duration of competition with the crop. Additionally, weed persistence and dominance are shaped by the crop type, climate and seasonal conditions, sowing date, and cultivation practices (Ramli et al., 2024).

Broadleaf weeds are a significant concern in rice fields due to their competitive nature and impact on crop yields. Broadleaf weeds frequently have a high seed production rate, allowing them to invade rice fields quickly, and their broad leaves produce extensive shade and can hinder the growth of rice seedlings. This characteristic makes it difficult to control their proliferation once established (Kumar et al., 2017). These weeds, such as *Monochoria vaginalis* and *Lindernia procumbens*, can dominate weed communities in rice fields, accounting for up to 60% of the total weed population in some areas. *Eclipta prostrata, Mimosa pudica*, and *Ludwigia hyssopifolia* compete aggressively with rice for essential resources. This competition can severely reduce rice yields if not correctly managed. In recent years, changes in rice cultivation practices, particularly the shift toward direct-seeded rice (DSR), have increased the prevalence of broadleaf weeds. The continuous use of grass-specific herbicides in DSR systems has allowed broadleaf weeds and sedges to become more dominant, leading to yield reductions of up to 52-64% (Patidar and Kaur, 2024; Chauhan, 2012).

According to Les (2020), Monochoria is a genus of obligate aquatic herbs that are either annual or facultatively perennial. These plants are well adapted to fluctuating water levels through heterophylly—they produce sessile, linear leaves when submerged and broad-bladed, petiolate leaves when floating or emergent. *Monochoria vaginalis* (MV), a broadleaf monocot weed common in wetland rice ecosystems, is widespread across major rice-growing areas but is generally absent in drier regions (Athira, 2019). Commonly known as pickerelweed, *M. vaginalis* is a significant weed in Asian rice fields, competing with rice for essential resources such as nutrients, light, and space (Zhu et al., 2012). It is considered one of the five most prevalent weeds in paddy fields in China (Zhou et al., 2021). This species exhibits rapid growth and is capable of causing up to 44% yield loss (Sujinah et al., 2022). The interference it causes in rice growth and development significantly reduces yield potential (Zhu et al., 2012). Although early-stage *M. vaginalis* seedlings can be managed with pre-emergence herbicides such as Butachlor, it remains a persistent threat and can cause substantial grain yield losses (Shuman-Goodier et al., 2021). Research has shown that severe infestations can lead to yield reductions of up to 55% (Zhu et al., 2012).

*Sphenoclea zeylanica* (SZ) Gaertn., commonly known as gooseweed, is an annual herbaceous aquatic weed belonging to the family Sphenocleaceae (Krumsri et al., 2020). It is widely distributed across tropical and warm-temperate regions and is native to the Eastern Hemisphere, including China, Southeast Asia, South Asia, Africa, and Madagascar (Carter et al., 2014). S. zeylanica thrives in both terrestrial and freshwater systems under tropical and warm climates (Vu et al., 2018). It reproduces mainly by seeds, producing large quantities, with most germinating between June and December (Carter et al., 2014). Reddy (2020) reported that the seeds remain viable even after two years of storage under favorable conditions, with germination rates of 6% and 34% at 17 and 48 days after sowing (DAS), respectively.

This weed typically emerges directly from seeds in puddled rice fields and spreads rapidly due to its high seed production capacity. Its adaptability to both waterlogged and moist conditions, including germination under submerged environments, makes it a serious challenge in rice cultivation (Reddy, 2020). The methanol extract of *S. zeylanica* has been found to suppress the shoot and root growth of several weed species such as *Leptochloa chinensis, Chloris barbata, Dactyloctenium aegyptium, Pennisetum pedicellatum, Pennisetum setosum, Hygrophila erecta, Mimosa invisa, Hyptis suaveolens*, and *Scirpus articulatus* (Krumsri et al., 2020). It is considered one of the world’s worst rice weeds, found in at least 28 countries, and is also a problem in cotton, wheat (especially in rice-wheat rotations), and soybean fields. This fast-growing annual weed can reduce rice yields by 45–50%, disrupt harvesting, and delay drying when its biomass remains green and succulent (Rojas-Sandoval, 2023).

*Limnocharis flava* (LF) is another problematic emergent aquatic weed. Native to tropical and subtropical America, it has become invasive in several Asian countries, including Malaysia, Indonesia, Thailand, Vietnam, Sri Lanka, and India (Nishan and George, 2018). It is a perennial herbaceous plant that can grow up to one meter in height (Zakaria et al., 2018). Belonging to the Limnocharitaceae (or Alismataceae) family, *L. flava* commonly invades irrigation canals and water systems, where it competes with native aquatic flora for light and nutrients, leading to ecological imbalance (Zakaria et al., 2021). It features glabrous stems and a scapigerous structure, with broad leaves that are rounded to ovate, sheathing with wavy edges and forming clusters above the water. The petioles are triangular with well-developed air spaces (Nishan and George, 2018; Putra et al., 2023). Although initially introduced as an ornamental species due to its attractive foliage and flowers, its aggressive growth and persistence have made it an invasive threat (Song et al., 2024).

In Malaysia and other parts of Southeast Asia, *Limnocharis flava* has become a serious weed in rice fields and irrigation systems. It has developed resistance to both synthetic auxin herbicides and acetohydroxyacid synthase (AHAS) inhibitors (Zakaria et al., 2021). According to Nishan and George (2018), direct-seeded rice is more affected by this weed than transplanted rice. The plant thrives on fertilized soils, grows rapidly, and has, in some cases, led to rice field abandonment due to uncontrollable infestations. It clogs irrigation channels and drainage systems, reducing water flow and leading to water stagnation in lower areas. During heavy rainfall, it further exacerbates crop damage by obstructing water outflow, causing field submergence (Seneviratne and Wijesundara, 2004; Nishan and George, 2018).

### 1.1 Hyperspectral imaging and machine learning for weed analysis

Precision weed management integrates cultural, mechanical, and chemical control methods to minimize herbicide use while reducing environmental impact. This balanced approach ensures effective weed control while protecting crop plantations (Agrawal, 2024; Monteiro and Santos, 2022). Precision weed control serves as a bridge between conventional chemical control and automated mechanical weed control, offering a more targeted and sustainable alternative. By identifying specific weed patches, herbicide application can be precisely localized, reducing unnecessary chemical use and improving efficiency. Accurate weed detection is essential for this strategy to be effective (Che'Ya, 2016).

Hyperspectral imaging (HSI) is an integration of modern imaging systems and traditional spectroscopy techniques (Sulaiman et al., 2022). It enables the acquisition of spectral information for each pixel in an image, providing a detailed spectral profile (Bhargava et al., 2024). While RGB imaging has demonstrated high performance in many weed detection applications, particularly for morphologically distinct weeds, its effectiveness can be limited during early growth stages of crop cultivation (Mensah et al., 2024). At these stages, rice plants and broadleaf weeds often share similar morphological and color characteristics, making them difficult to separate based on RGB features alone. Hyperspectral imaging (HSI) provides hundreds of contiguous spectral bands, enabling detection of subtle differences in pigment composition, water content, and internal leaf structure that are not visible to RGB sensors (Pérez-Ortiz et al., 2016). This enhanced spectral resolution allows for more accurate species‐level discrimination under challenging conditions and supports earlier, more targeted weed management interventions.

Machine learning (ML) methods offer a variety of classification algorithms for analyzing remote sensing imagery and have proven effective in identifying unwanted species using remote sensing (Papp et al., 2021). Generally, the ML framework involves learning from ‘experience’, known as training data, to execute the classification, regression, or clustering tasks. These training data are usually regarded as a feature described by a set of attributes or variables. The machine learning model works by predicting the pattern and trend of future events in crop monitoring and assessment (Zhang et al., 2019). The ML model’s performance in a particular task is evaluated by performance metrics that improve with experience over time. As a result, classification techniques have been a prominent research trend in machine learning for many years, informing various studies. This method seeks to create features from the input data. Furthermore, it is highly field-specific and requires significant human effort, leading to deep learning techniques (Alzubaidi et al., 2021).

### 1.2 Supervised classification model training

#### 1.2.1 Support vector machine

Support Vector Machine (SVM) is a robust classification algorithm that aims to find the optimal hyperplane that best separates data points of different classes. It is particularly effective in high-dimensional spaces and can handle both linear and non-linear classification problems by utilizing kernel functions. The SVM algorithm enhances classification accuracy by maximizing the margin between different class boundaries, contributing to robust generalization in the spectral classification of plant species (Ballanti et al., 2016).

#### 1.2.2 Minimum distance

The Minimum Distance classifier is a simple yet effective algorithm that calculates the spectral distance between each pixel and the mean spectral signature of each class. It assigns pixels to the class with the smallest distance, making it suitable for datasets with well-separated class means (Rose and Seldev, 2014). While less sophisticated than SVM, it offers faster processing times and can be advantageous in scenarios where spectral variability within classes is low.

#### 1.2.3 Parallelepiped

The Parallelepiped classifier uses a set of spectral thresholds defined by the minimum and maximum values of each class’s training data. Pixels falling within the defined spectral “box” are classified accordingly (De Moraes, 2004). This method is computationally efficient and helpful when the spectral ranges of classes are well-defined. However, its reliance on strict boundaries may lead to misclassification in cases of spectral overlap or high variability within classes.

The Parallelepiped classifier uses a set of spectral thresholds defined by the minimum and maximum values of each class’s training data. Pixels falling within the defined spectral “box” are classified accordingly. This method is computationally efficient and helpful when the spectral ranges of classes are well-defined. However, its reliance on strict boundaries may lead to misclassification in cases of spectral overlap or high variability within classes.

By using UAV-based hyperspectral imaging combined with a machine learning technique, this study aims to enhance the detection and classification of the broadleaf weeds (BLW) in rice fields at an early growth stage (Vegetative stage). The selected broadleaf weed species used in this study were *Monochoria vaginalis, Limnocharis flava*, and *Sphenoclea zeylanica*. The selection of these species of weeds as the focus of this study was based on their prevalence and significant impact in the study area. The study leverages ENVI Classic 5.3 software for supervised classification, where regions of interest (ROIs) are selected for training the classifier to recognize the spectral signatures of the three targeted weed species and the rice plants. Ultimately, the goal of this research is toward:

- Implements and compares multiple supervised classification methods (SVM, Minimum Distance, Parallelepiped) to identify the optimal algorithm for early-stage weed discrimination.
- Demonstrates the feasibility of UAV-HSI for scalable, non-destructive, high-accuracy weed mapping, which can reduce herbicide use and support sustainable rice production.

## 2 Methods and procedure

### 2.1 Study site

The experiment for this study was carried out in a rice field near Pusat Benih Padi Felcra Sdn Bhd (Perak) managed by FELCRA Plantation Services Sdn. Bhd. is located in Bandar Seberang Perak, Kampung Gajah, Perak, Malaysia (postcode 36800). The region’s latitude and longitude are roughly 4.1263° N and 100.9789° E. Block L5A, plot 121 was chosen for the experiment site due to its proximity to the central irrigation canal and the high weed pressure. This particular rice field location is noted for its good soil and favorable environment for rice production. The crop cultivation management program followed the FELCRA procedure which is based on the Rice Check Padi Edisi 2022 guidelines issued by the Department of Agriculture (DOA), Malaysia (Department of Agriculture Malaysia (DOA), 2022). The place has a tropical rainforest climate with consistently high humidity and temperatures throughout the year. The average annual rainfall is approximately 2000–2500 mm, promoting rice farming while creating an environment suitable for weed development. This region’s soil is alluvial mainly, nutrient-rich, and capable of retaining water. These traits are crucial for rice farming, but they also promote the growth of many weeds.

**Figure 1:**
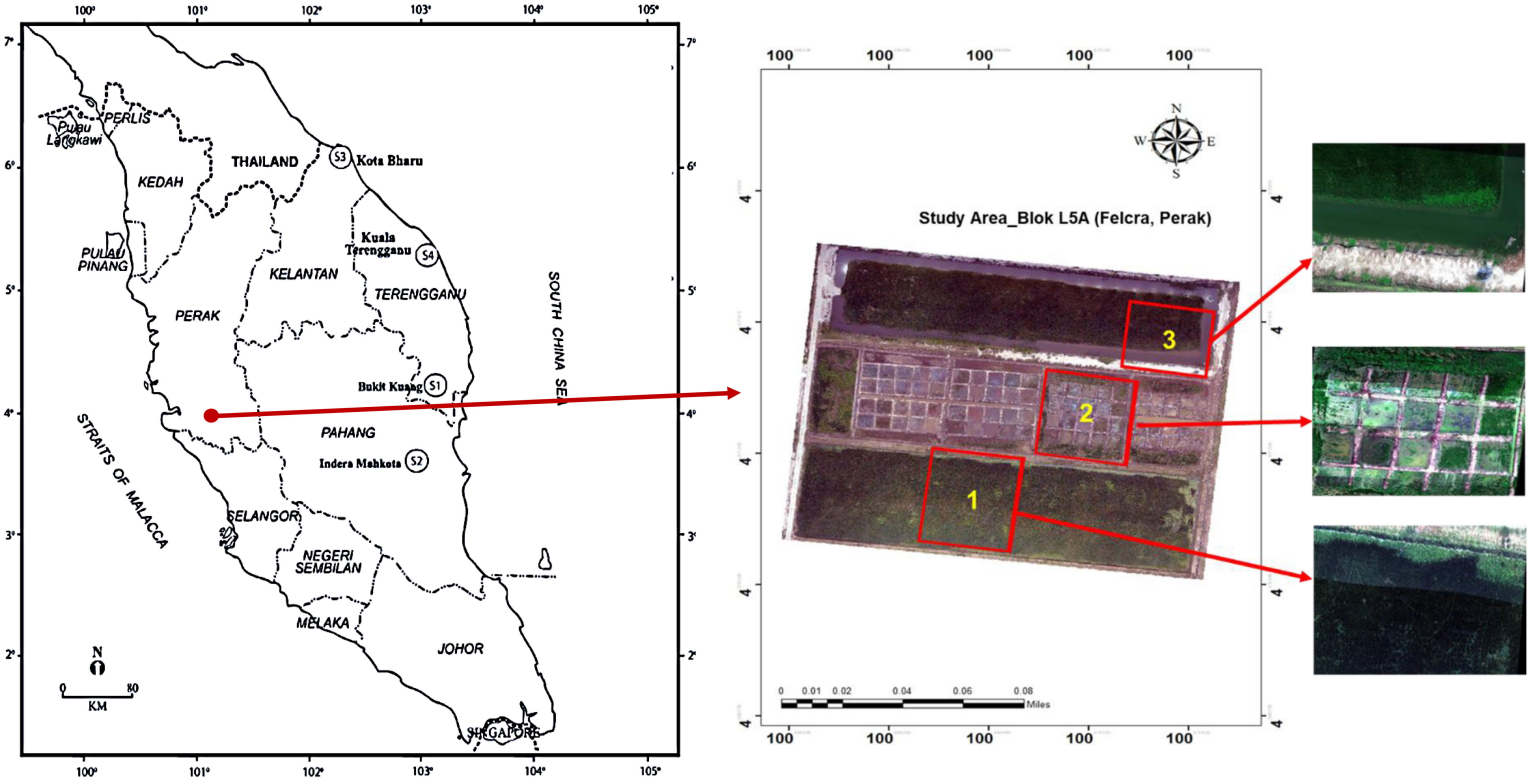
Study site for broadleaf weed in rice field at Felcra Berhad (Perak).

Weed analysis plots were established in three different areas, selected based on preliminary surveys assessing the density of the target broadleaf weed species; *Limnocharis flava* (LF), *Monochoria vaginalis* (MV), and *Sphenoclea zeylanica* (SZ). Density assessments were conducted using hyperspectral image analysis to identify plots with adequate representation of each species. Details of the study plots are described below (**Figure 1**; **Table 1**).

**Table 1:** Info on study Plot at Blok l5A, Felcra Perak.

No.	Plot	Broadleaf weed on focus	Plot area (m^2)^
1.	Plot 1	*Limnocharis flava*	2300
2.	Plot 2	*Monochoria vaginalis*	2500
3.	Plot 3	*Sphenoclea zeylanica*	2200

### 2.2 Acquisition of hyperspectral imagery

The image acquisition procedure for hyperspectral data collection involves three main stages: preparation and calibration, field operation, and data retrieval. Hyperspectral data was collected by using a UAV (DJI Matrice 600) and the hyperspectral camera, Resonon Pika L (captures data in the 400–1000 nm spectral range with 281 spectral bands). The UAV-based imaging system includes (i) a Resonon Pika L hyperspectral camera (Spectronon Pro, Resonon, Bozeman, MT); (ii) visible-near infrared (V-NIR) objective lenses for the Pika L camera with a focal length of 23 mm, field of view (FOV) of 13.1 degrees, and instantaneous field of view (IFOV) of 0.52 mrad; and (iii) a global positioning system (GPS) and the inertial measurement unit IMU (DJI) flight control system for multi-rotor aircraft, to record sensor position and orientation. Data was collected at 40 meters above the ground.

Time of Day (TOD) refers to the specific period selected for aerial imaging, taking into account lighting conditions, sun angle, and shadows, all of which influence the quality of captured images (Barbosa Júnior et al., 2022). Although research on the direct effects of TOD is limited, the most commonly recommended imaging windows are between 9:00 a.m. and 2:00 p.m., 10:00 a.m. and 3:00 p.m., or 11:00 a.m. and 2:00 p.m (Bongomin et al., 2024; Gonçalves et al., 2022; Mao et al., 2021). These timeframes align with optimal sunlight conditions, providing stable lighting and minimal atmospheric disturbances, which are critical for obtaining high-quality spectral data in vegetation analysis. Proper TOD selection helps ensure consistent and accurate remote sensing measurements, reducing errors caused by shadows or variable light intensity. Data collection schedule is described in **Table 2**.

**Table 2:** Data collection schedule.

Venue: rice field (Felcra Sdn Bhd, Perak)			
No.	Date	Day after sowing (DAS)	Rice crop stage
1.	05/10/2023	0	Sowing day
2.	20/10/2023	15DAS	Stage 1: Vegetative • Germination • Seedling emergence
3.	30/10/2023	25DAS	Stage 1: Vegetative • 2^nd^ leaf – 5^th^ leaf (1^st^ tiller)
4.	05/11/2023	30DAS	Stage 1: Vegetative • Tillering

The camera was mounted on a DJI Ronin-MX gimbal, and the imager was activated via a Resonon flight computer connected to a GPS receiver, using a target map generated in Google Earth. During flight, the Resonon Pika L sensor captured hyperspectral images, while GPS coordinates and flight metadata were recorded for accurate georeferencing. Data was stored on the sensor’s onboard system or transferred in real time to the ground control station. After landing, the hyperspectral data was retrieved, backed up, and prepared for analysis to ensure accuracy and reliability in weed detection.

The hyperspectral raw data obtained are spectrally calibrated but are not corrected for the illumination functions, so the result cannot be directly interpreted. The image capture from the hyperspectral sensor was pre-processed using Spectronon Pro (Resonon). The spectrum of the image will be used to distinguish the spectral signature between rice plants and the broadleaf weeds. The spectral signature was used to identify the selected broadleaf weed species in rice crops for real-time detection of the broadleaf weeds in the rice fields. The region of interest was chosen randomly from the developed 2D maps, and the files were saved in KML format.

### 2.3 Hyperspectral imagery processing workflow

**Figure 2:**
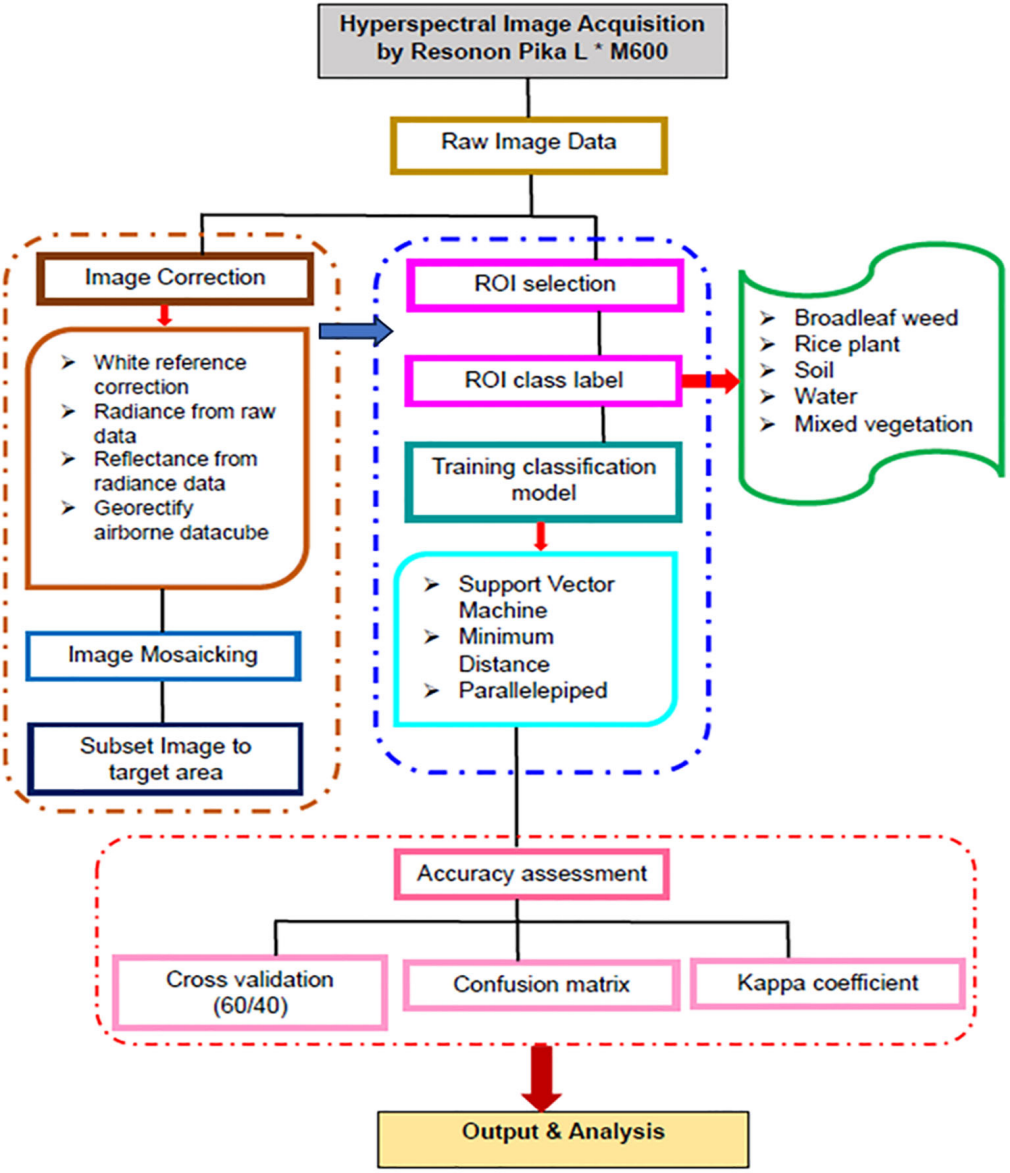
Methodology flow chart for hyperspectral image supervised classification analysis.

The methodology flow chart of weed detection analysis is described as **Figure 2** below. First step in the supervised classification process involves preprocessing the hyperspectral data. This is to ensures the data quality and prepares it for further analysis. After completing image correction and image mosaicking in SpectrononPro, the image data can be subset to target area in Envi Classic 5.3.

#### 2.3.1 Image calibration correction

**Figure 3:**
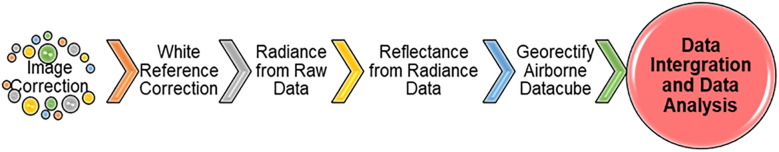
Image calibration correction process flow.

The process of image correction for hyperspectral images captured using the Resonon Pika L sensor involves several essential steps to ensure the data’s accuracy and usability for analysis, particularly for vegetation and weed detection in rice fields (**Figure 3**). This pre-processing procedure is run using SpectrononPro software.

i. White Reference Correction: This step involves calibrating the sensor to account for varying lighting conditions and sensor sensitivity. A white reference panel, which has a uniform reflectance spectrum, is used during the data acquisition process. By normalizing the hyperspectral data against the white reference, variations caused by lighting inconsistencies or sensor noise are minimized. Denoising techniques improve signal-to-noise ratio and classification accuracy in hyperspectral imagery, making them a valuable preprocessing step for improved analysis (Rasti et al., 2018).
ii. Radiance from Raw Data: Intermediate-form hyperspectral image data can be processed directly or converted into radiance values and estimates of signal-dependent noise, benefiting image analysis (Skauli, 2009). The raw data acquired by the hyperspectral sensor represents digital counts. These digital counts are converted into radiance values, which express the actual energy measured by the sensor in physical units. This conversion accounts for the sensor’s calibration factors and the spectral response, ensuring that the data is meaningful for further analysis.
iii. Reflectance from Radiance Data and Spectrally Flat Reference Spectrum: Hyperspectral image exploitation algorithms require reflectance spectra retrieved from observed radiance spectra (Golowich et al., 2018). Radiance data is then converted into reflectance values, which are crucial for identifying vegetation features. This is done by dividing the radiance data by the radiance of the spectrally flat reference (e.g., the white reference panel) captured during the calibration process. Reflectance values eliminate the effects of varying light intensity and are used to derive the actual spectral signatures of the target vegetation and weeds.
iv. Georectify Airborne Datacube: The hyperspectral data, captured in the form of a hypercube (spatial and spectral dimensions), is georectified to align with real-world geographic coordinates. This involves integrating GPS and inertial measurement unit (IMU) data collected during the flight. The hyperspectral mapping system, which includes a pushbroom spectrometer, photogrammetric camera, and GPS-INS, can georectify pushbroom data fully automatically for agricultural mapping and monitoring applications (Suomalainen et al., 2014).
v. Data Analysis: Advanced data analysis techniques, such as machine learning or spectral unmixing, are applied to the corrected data. This step identifies spectral signatures unique to weeds and crops, enabling accurate classification and mapping of vegetation. This systematic image correction process ensures that hyperspectral images are accurate, reliable, and ready for use in weed detection and vegetation analysis.

#### 2.3.2 Region of interest selection

In the weed detection analysis using ENVI Classic 5.3, the Region of Interest (ROI) selection is a crucial step in the supervised classification process. The ROI represents specific areas within the hyperspectral image that serve as samples for training and validating the classification algorithm. For this study, the ROI classes included (**Table 3**):

**Table 3:** ROI set for image analysis.

No.	Color	Class
1.	Red	Broadleaf weed (*Limnocharis flava, Monochoria vaginalis*, and *Sphenoclea zeylanica)*
2.	Green	Rice plant
3.	Purple	Mixed vegetation (area containing a combination of plant species)
4.	Blue	Water (visible water bodies or waterlogged area)
5.	Yellow	Soil (bare soil regions)

The ROI creation process involved preparing the hyperspectral image and utilizing the ROI tool in ENVI Classic 5.3 to manually or semi-automatically select representative regions. These regions were labeled according to their respective classes, ensuring a diverse and accurate representation of the study area (refer to **Table 4**).

**Table 4:** Number of ROI created for hyperspectral image supervised classification analysis.

No.	Cycle/DAS	Class	No. of polygon (ROI)	No. of training ROI (60%)	No. of validation ROI (40%)	Total pixel count
Plot 1: *Limnocharis Flava*						
1.	Cycle 1/ 15DAS	BLW_LF	60	36	24	653
2.		Rice Plant	70	42	28	954
3.		Water	20	12	8	109
4.		Soil	20	12	8	90
5.		Mixed Vegetation	20	12	8	69
6.	Cycle 2/ 25DAS	BLW_LF	60	36	24	463
7.		Rice Plant	60	36	24	750
8.		Water	20	12	8	53
9.		Soil	20	12	8	77
10.		Mixed Vegetation	20	12	8	88
11.	Cycle 3/ 30DAS	BLW_LF	40	24	16	280
12.		Rice Plant	50	30	20	560
13.		Water	15	9	6	109
14.		Soil	15	9	6	43
15.		Mixed Vegetation	15	9	6	42
Plot 2: *Monochoria vaginalis*						
16.	Cycle 1/ 15DAS	BLW_MV	35	21	14	141
17.		Rice Plant	65	39	26	270
18.		Water	20	12	8	110
19		Soil	30	18	12	191
20.		Mixed Vegetation	20	12	8	88
21.	Cycle 2/ 25DAS	BLW_MV	30	18	12	254
22.		Rice Plant	35	21	14	306
23.		Water	20	12	8	268
24.		Soil	20	12	8	195
25.		Mixed Vegetation	20	12	8	172
26.	Cycle 3/ 30DAS	BLW_MV	35	21	14	377
27.		Rice Plant	35	21	14	397
28		Water	15	9	6	181
29.		Soil	15	9	6	299
30.		Mixed Vegetation	15	9	6	241
Plot 3: *Sphenoclea zeylanica*						
31.	Cycle 1/ 15DAS	BLW_SZ	35	21	14	114
32.		Rice Plant	40	24	16	204
33.		Water	20	12	8	149
34.		Soil	20	12	8	178
35.		Mixed Vegetation	20	12	8	114
36.	Cycle 2/ 25DAS	BLW_SZ	40	24	16	329
37.		Rice Plant	50	30	20	357
38.		Water	25	15	10	198
39.		Soil	25	15	10	302
40.		Mixed Vegetation	20	12	8	175
41.	Cycle 3/ 30DAS	BLW_SZ	50	30	20	519
42.		Rice Plant	45	27	18	874
43.		Water	25	15	10	705
44.		Soil	25	15	10	644
45.		Mixed Vegetation	20	12	8	340

A critical aspect of the ROI strategy was the distribution of samples into 60% for training and 40% for validation (Sivakumar et al., 2024). The training samples were used to “teach” the classification algorithm, providing known spectral signatures for each class. Meanwhile, the validation samples, which were withheld from the training process, were used to assess the model’s accuracy and its ability to generalize to new, unseen data (Xu and Goodacre, 2018).

#### 2.3.3 Classification execution, validation, and accuracy assessment

Once the training data (ROIs) and algorithm are selected, the classification is executed to assign each pixel in the hyperspectral imagery to one of the predefined classes. ENVI Classic 5.3 generates a classified image in which each pixel is color-coded according to its assigned class. Validation ensures the reliability of the classification results. ENVI Classic 5.3 provides tools for quantitative accuracy assessment:

- Confusion Matrix: Compares the classified image with ground truth data, providing metrics like overall accuracy, user’s accuracy (UA), and producer’s accuracy (PA) (as described in **Table 5**).
- Kappa Coefficient: Measures the agreement between classification results and ground truth, accounting for chance agreement.

**Table 5:** The definition of UA and PA (Congalton, 1991).

Metric	Definition	Formula
User’s Accuracy (UA)	Probability that a pixel classified into a given class represents that class on the ground (reliability).	$UA=\frac{correctly\;classified\;pixel\;in\;class}{Total\;pixels\;classified\;as\;that\;class}\times 100\;\;$
Producer’s Accuracy (PA)	Probability that a pixel of a given class in the reference data has been correctly classified (completeness).	$PA=\frac{correctly\;classified\;pixel\;in\;class}{Total\;reference\;pixels\;for\;that\;class}\times 100\;\;$

These metrics provide a quantitative evaluation of the classification performance, allowing researchers to identify and address potential shortcomings in the workflow. Performance metrics such as precision, recall, and F1-score are also computed to assess the effectiveness of the classification model.

## 3 Results and analysis

This analysis evaluates the classification performance of three supervised classification techniques—Support Vector Machine (SVM), Minimum Distance (MD), and Parallelepiped (PP)—for distinguishing between the selected Broadleaf Weed, Rice Plant, Mixed Vegetation, Soil, and Water at 15 DAS, 25 DAS, and 30 DAS using ENVI Classic 5.3.

### 3.1 Detection of broadleaf weed: *Limnocharis flava*

**Figure 4:**
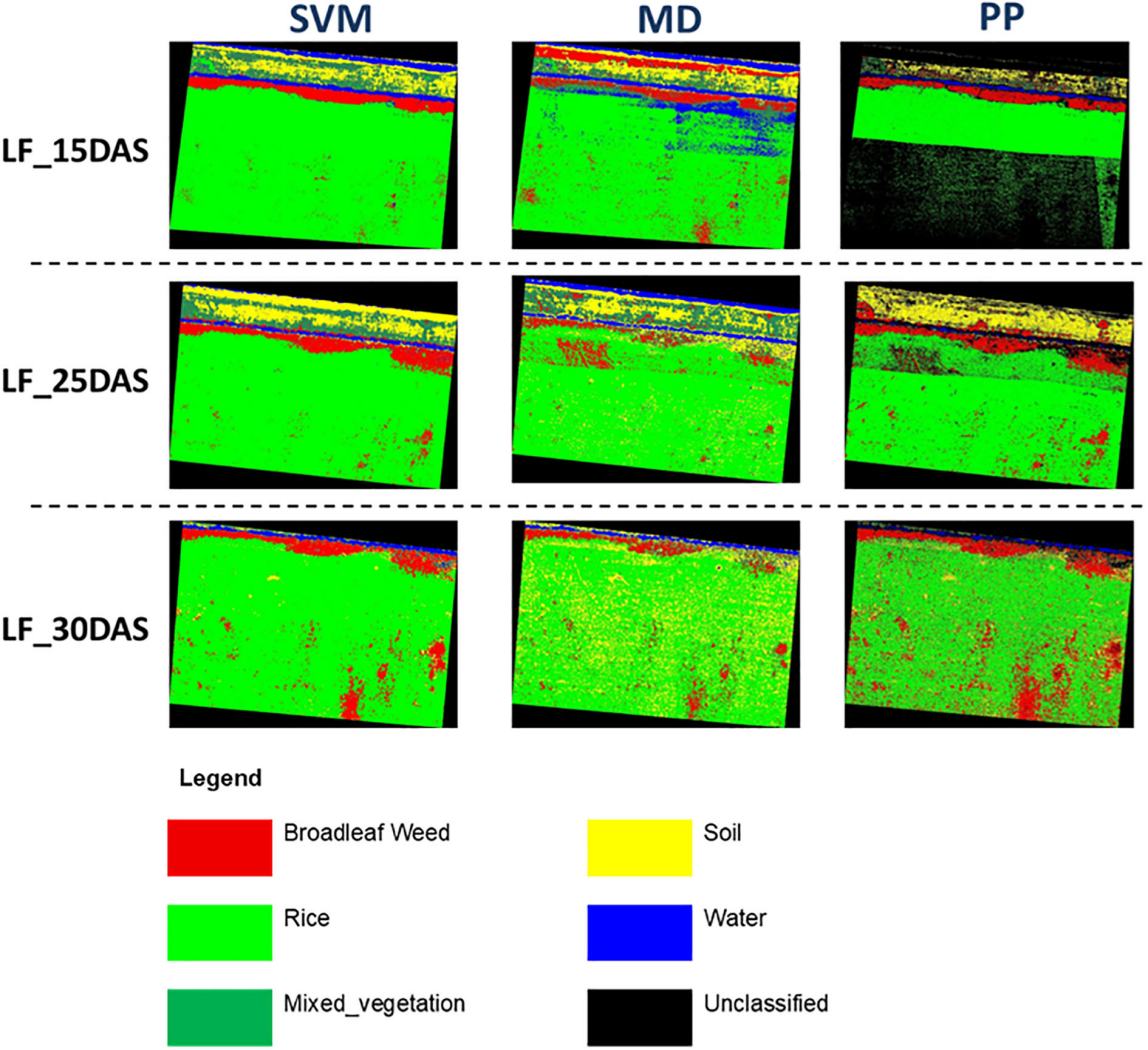
LF detection in rice field implementing supervised classification algorithm.

The classification performance for *Limnocharis flava* (Blw_Lf) using UAV-based hyperspectral imagery across different growth stages shows a clear trend in favor of the Support Vector Machine (SVM) classifier (**Figure 4**). At 15 DAS (**Table 6**), SVM already achieves a high producer accuracy (90.85%) and user accuracy (99.85%), indicating it is highly capable of identifying and correctly labeling LF even at an early stage. In contrast, the Minimum Distance method provides lower performance with 69.86% producer accuracy and 93.65% user accuracy. In comparison, Parallelepiped yields better producer accuracy (77.95%) but a much lower user accuracy (35.87%), suggesting high misclassification of other classes as LF.

**Table 6:** Comparison of producer accuracy (PA) and user accuracy (UA) for each algorithm for plant classification (*Limnocharis flava*) at 15, 25, and 30DAS.

Classifier/ROI class	SVM		MD		PP	
Class	Prod. acc. (%)	User acc. (%)	Prod. acc. (%)	User acc. (%)	Prod. acc. (%)	User acc. (%)
PA and UA at 15DAS						
Blw_LF	99.85	99.85	85.3	98.76	83.46	97.15
Rice	100	99.9	88.68	99.65	79.35	99.87
Water	100	100	100	73.65	81.65	100
Soil	100	100	100	94.74	84.44	100
Mixed Vegetation	97.1	98.53	91.3	28.77	53.62	92.5
PA and UA at 25DAS						
Blw_LF	100	99.78	80.78	78.08	85.96	87.09
Rice	100	100	84.53	98.45	81.07	100
Water	100	100	100	88.33	58.49	100
Soil	100	100	77.92	66.67	90.91	93.33
Mixed Vegetation	98.86	100	90.91	50.63	48.86	100
PA and UA at 30DAS						
Blw_LF	99.29	99.64	87.14	99.59	83.57	90
Rice	100	98.42	81.96	95.43	73.04	99.51
Water	100	100	100	98.2	65.14	100
Soil	79.07	100	62.79	19.57	81.4	39.33
Mixed Vegetation	100	97.67	85.71	61.02	78.57	89.19

At 25 DAS (**Table 6**), SVM further improves, achieving perfect scores (100%) in both producer and user accuracy for Blw_Lf, which reflects its robustness and reliability. Minimum Distance also shows improved results (80.78% PA and 78.08% UA), and Parallelepiped performs moderately better (85.96% PA, 87.09% UA), but both still lag behind SVM. By 30 DAS, SVM maintains its high accuracy, with a producer accuracy of 99.29% and user accuracy of 99.64%. Minimum Distance also performs well (87.14% PA and 94.15% UA), while Parallelepiped records good results (83.57% PA, 87.67% UA), though still not as consistently accurate as SVM.

#### 3.1.1 Comparative analysis of classification models for LF detection

**Table 7** presents the classification performance of SVM, MD, and PP classifiers for detecting *Limnocharis flava* at three different growth stages: 15 Days After Sowing (DAS), 25 DAS, and 30 DAS. The analysis is based on Overall Accuracy, Kappa Coefficient, Mean, and Standard Deviation (StDev).

**Table 7:** Classification analysis of LF at 15DAS, 25DAS, 30DAS.

Species/DAS	*Limnocharis Flava*_15DAS				*Limnocharis Flava*_25DAS				*Limnocharis Flava*_30DAS			
Classifier	Overall accuracy	Kappa coefficient	Mean	StDev	Overall accuracy	Kappa Coefficient	Mean	StDev	Overall accuracy	Kappa coefficient	Mean	StDev
Support Vector Machine	99.84	0.99	2.9	1.6	99.96	0.99	3.11	1.72	99.26	0.99	2.68	1.59
Minimum Distance	91.02	0.88	3.02	1.65	89.81	0.86	3.26	1.7	84.62	0.77	2.58	0.92
Parallelepiped	80.213	0.71	3.02	1.65	87.54	0.83	3.03	1.65	75.63	0.65	1.54	1.24

SVM consistently demonstrates the highest classification performance across all time points. The overall accuracy remains above 99%, with a Kappa coefficient close to 1.0, indicating excellent classification agreement. The mean values range between 2.68 and 3.11, with relatively low standard deviation (StDev), suggesting that SVM maintains high stability and precision throughout different growth stages.

At 15 DAS, the overall accuracy is 99.84%, with a Kappa coefficient of 0.9974, indicating a nearly perfect classification. The accuracy further improves slightly at 25 DAS (99.96%), and remains very high at 30 DAS (99.26%). The slight variations in standard deviation suggest that SVM maintains a consistent and robust classification, making it the most reliable model for spectral classification of *Limnocharis flava*.

MD performs moderately well but exhibits lower classification accuracy than SVM. The overall accuracy drops from 91.02% (15 DAS) to 89.81% (25 DAS) and further declines to 84.62% (30 DAS). Similarly, the Kappa coefficient declines from 0.8783 at 15 DAS to 0.77 at 30 DAS, indicating increased misclassification at later growth stages. The mean values fluctuate between 2.58 and 3.25, with a higher standard deviation compared to SVM, implying that MD is more prone to spectral variability. The decline in accuracy at 30 DAS suggests that MD struggles with classifying mixed vegetation and soil, possibly due to increased spectral complexity as the plants mature.

PP shows the weakest classification performance, with significantly lower accuracy and higher variability. The overall accuracy starts at 80.21% at 15 DAS, drops to 87.54% at 25 DAS, and declines further to 75.63% at 30 DAS. The Kappa coefficient follows a similar trend, dropping from 0.711 to 0.65, indicating substantial classification errors and increased misclassification.

The mean values are consistently lower (between 1.54 and 3.03), and the standard deviation is higher compared to SVM, highlighting that PP lacks robustness and stability. The lower accuracy at 30 DAS suggests that PP struggles significantly with separating *Limnocharis flava* from other vegetation and soil.

##### 3.1.1.1.1 Vegetation cover analysis in rice _LF

**Figure 5:**
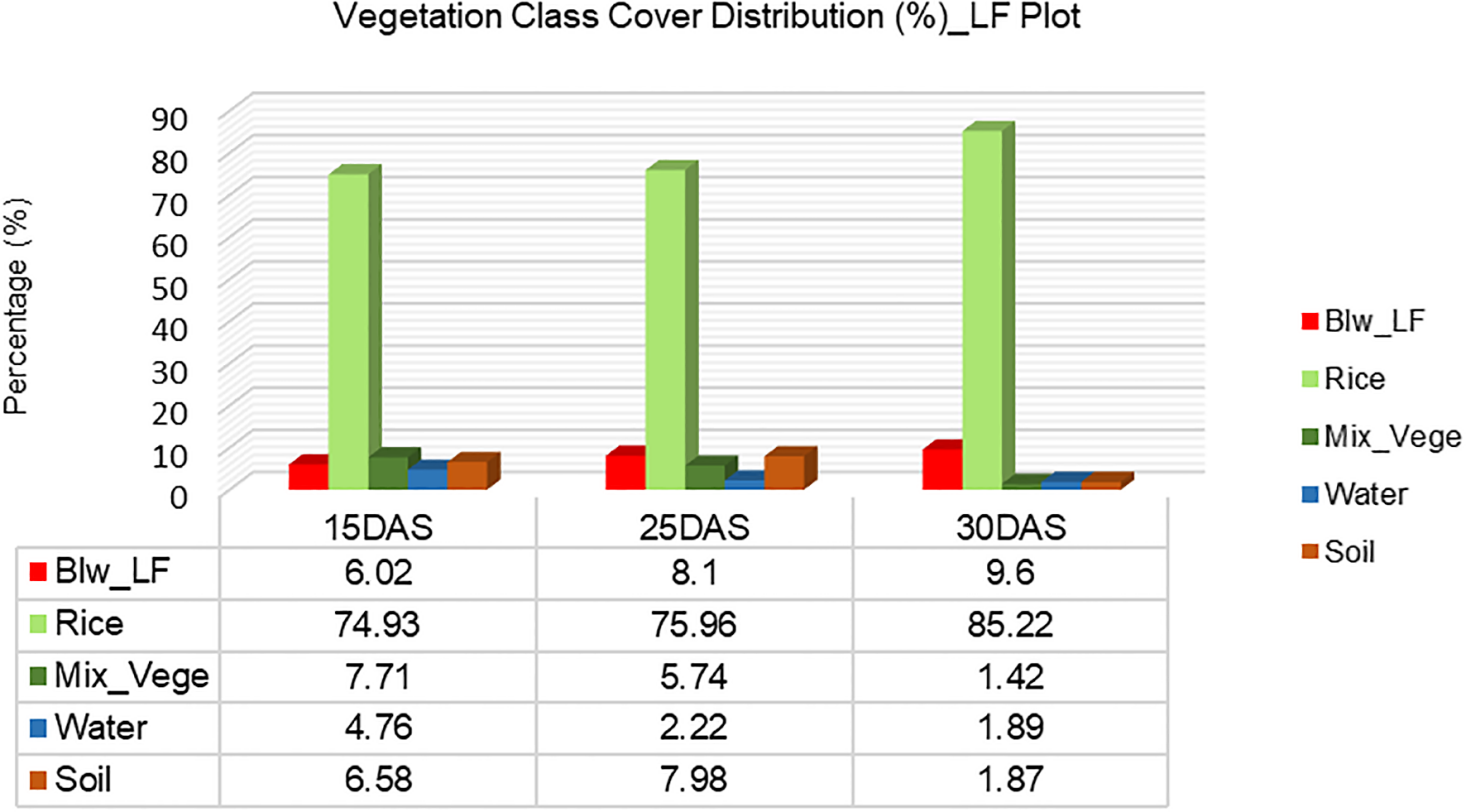
Vegetation class cover distribution (%) for Plot 1_LF.

The vegetation cover analysis in the rice field, based on SVM classification, provides key insights into the dynamics of different land cover categories—BLW_LF, Rice, Mixed Vegetation, Water, and Soil—at three growth stages: 15 Days After Sowing (DAS), 25 DAS, and 30 DAS (**Figure 5**). The first chart illustrates the percentage distribution of vegetation cover across these time points, while the second chart highlights the rate of change in cover percentage from 15 DAS to 30 DAS.

**Figure 6:**
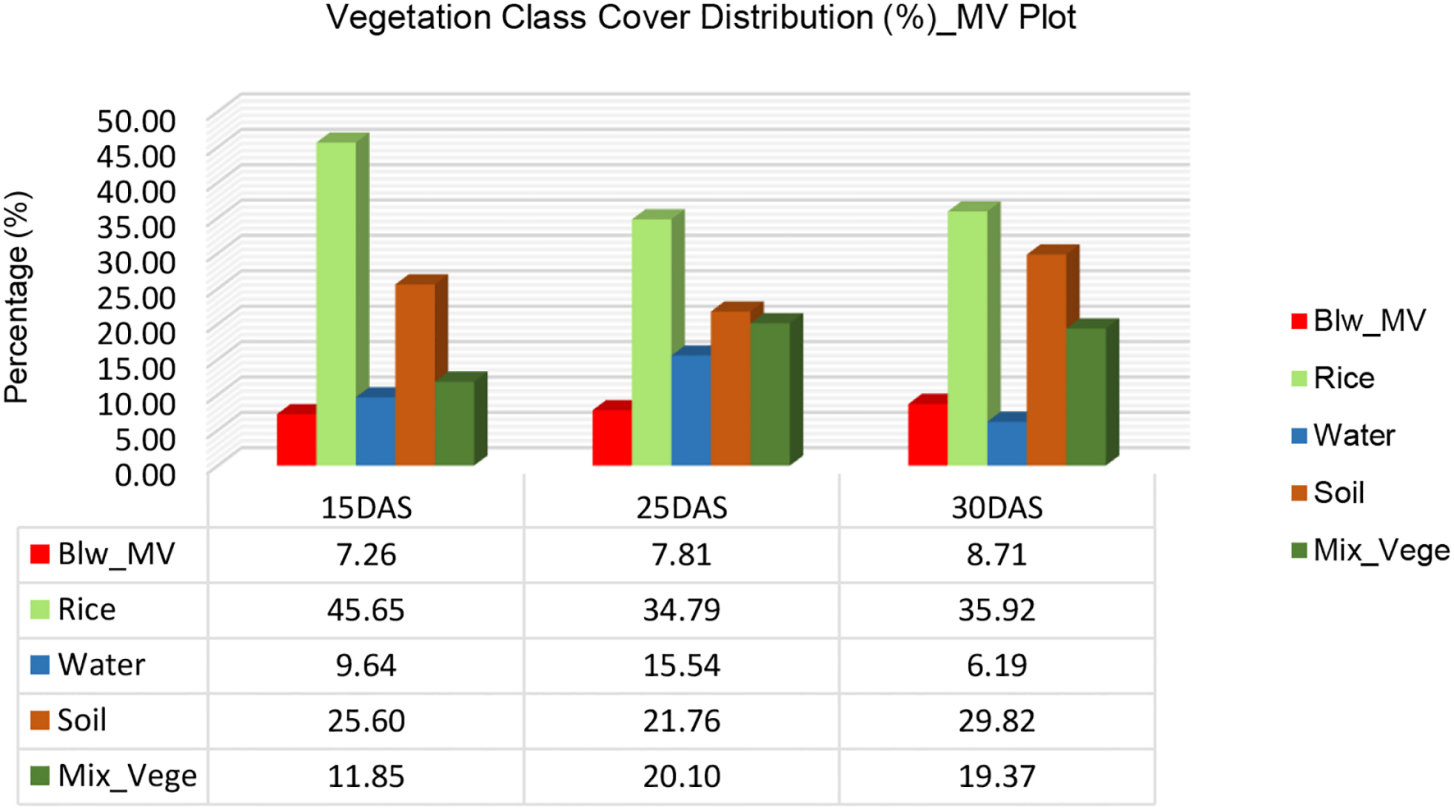
Vegetation class cover distribution (%) for Plot 2_MV.

The results in **Figure 6** indicate that rice vegetation exhibited the most significant increase over time, starting at 74.93% at 15 DAS, rising slightly to 75.96% at 25 DAS, and reaching a peak of 85.22% at 30 DAS. This 10.29% increase in rice coverage suggests successful crop establishment and dominance in the field, likely suppressing competing plant species. In contrast, broadleaf weed (BLW_LF) presence increased gradually, from 6.02% at 15 DAS to 8.1% at 25 DAS, and further to 9.6% at 30 DAS. This 3.58% increase implies that while rice growth is dominant, some weed expansion still occurs, highlighting the need for early weed management interventions.

**Figure 7:**
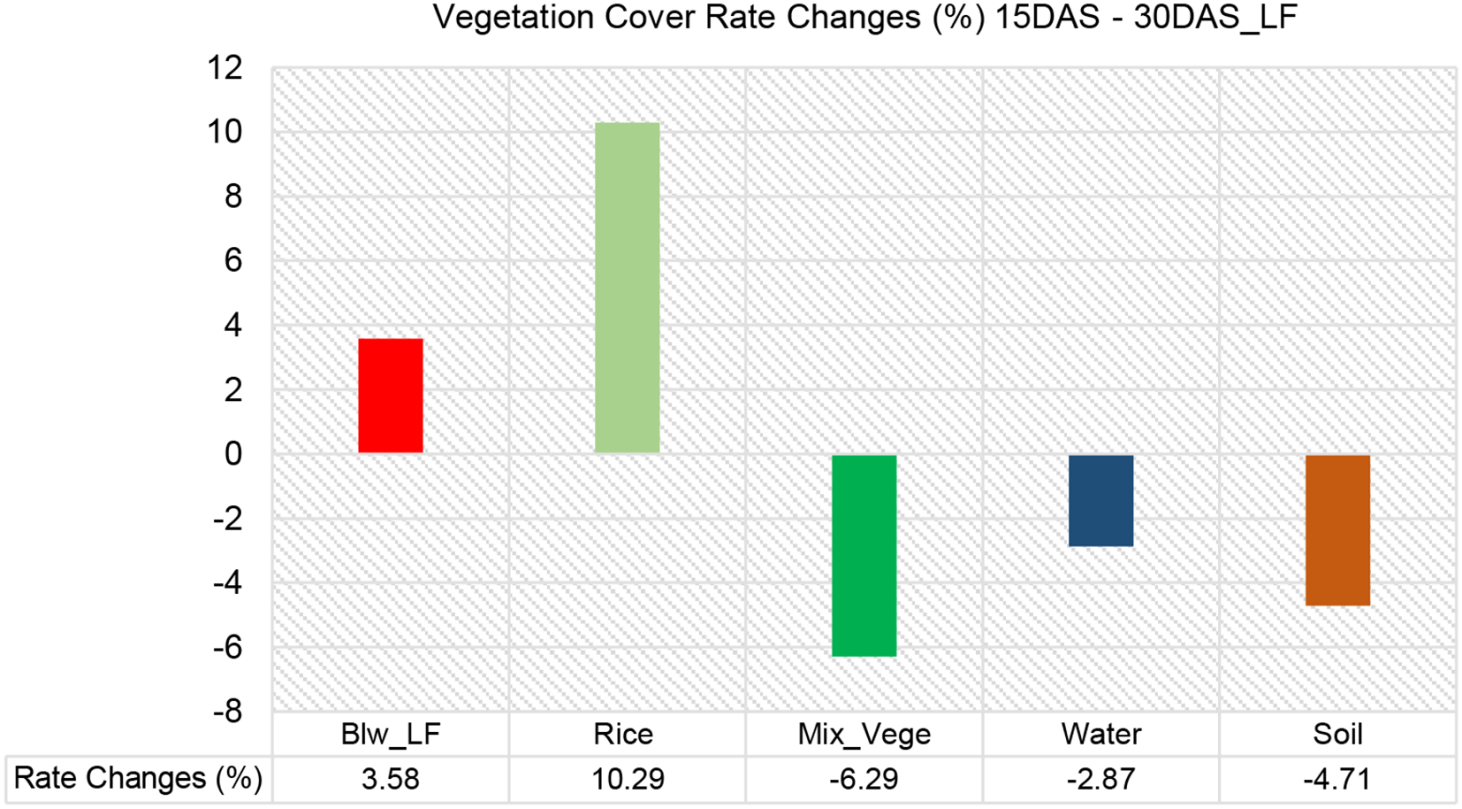
Vegetation cover rate changes (%) for LF in 15DAS – 30DAS.

A significant trend observed is the drastic reduction in mixed vegetation coverage, which declined from 7.71% at 15 DAS to 5.74% at 25 DAS, and finally to just 1.42% at 30 DAS (**Figure 7**). This 6.29% decrease indicates that as rice plants grow and canopy coverage expands, mixed vegetation species are either suppressed, absorbed into other classes, or outcompeted. A similar downward trend is seen in water and soil coverage. Water coverage shrank from 4.76% at 15 DAS to 2.22% at 25 DAS, and further to 1.89% at 30 DAS, reflecting potential field drainage, reduced irrigation, or increasing vegetation coverage over previously exposed water surfaces. Likewise, soil exposure increased slightly between 15 DAS (6.58%) and 25 DAS (7.98%), but then sharply declined to 1.87% at 30 DAS, suggesting that as rice and weeds grow, they effectively cover the bare soil, reducing its visibility in hyperspectral classification.

From a comparative perspective, rice exhibited the highest rate of positive change (+10.29%), followed by broadleaf weeds (+3.58%), while mixed vegetation (-6.29%), water (-2.87%), and soil (-4.71%) all experienced declines. The increase in rice dominance and reduction in other classes indicates that proper crop establishment can effectively outcompete weeds and other vegetation types, but the persistent increase in broadleaf weeds suggests the need for improved weed management strategies to minimize competition for nutrients and space.

### 3.2 Detection of broadleaf weed: *Monochoria vaginalis*

**Figure 8:**
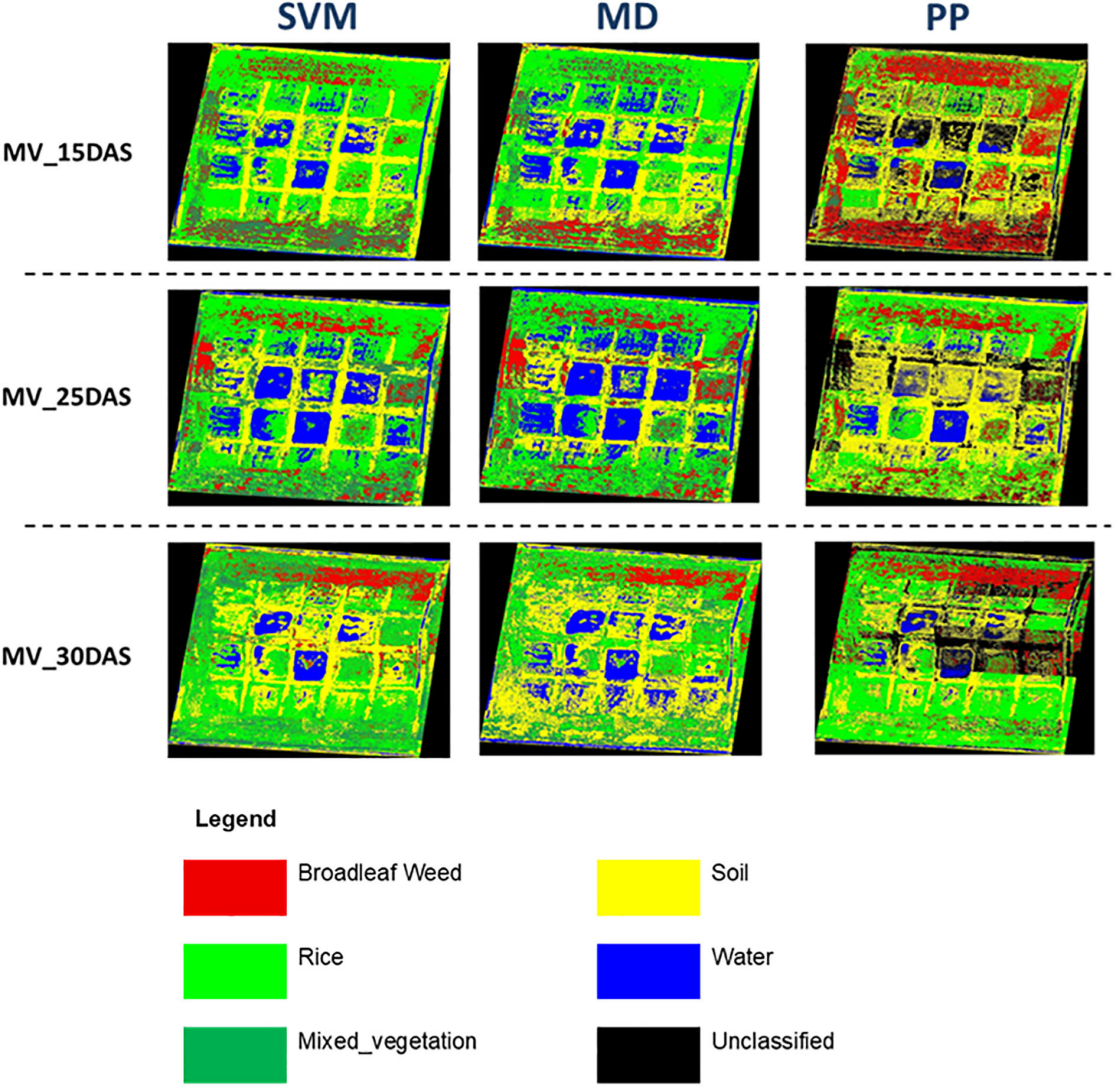
MV detection in a rice field implementing a supervised classification algorithm.

The classification results for *Monochoria vaginalis* (MV) using three algorithms, which are SVM, MD, and PP, at three different growth stages (15, 25, and 30 DAS) demonstrate varying levels of accuracy in hyperspectral image analysis (**Figure 8**). Overall, SVM consistently outperforms the other methods across all stages. At 15 DAS (**Table 8**), SVM achieves high producer accuracy (92.91%) and user accuracy (97.04%) for Blw_MV, indicating excellent detection and reliability. In contrast, Minimum Distance shows lower producer accuracy (63.12%) and user accuracy (77.39%), while Parallelepiped performs moderately in producer accuracy (84.40%) but poorly in user accuracy (39.27%), suggesting a high rate of false positives.

**Table 8:** Comparison of producer accuracy and user accuracy for each algorithm for plant classification (*Monochoria vaginalis*) at 15, 25, and 30DAS.

Classifier/ROI class	SVM		MD		PP	
Class	Prod. acc. (%)	User acc. (%)	Prod. acc. (%)	User acc. (%)	Prod. acc. (%)	User acc. (%)
PA and UA at 15DAS						
Blw_MV	92.91	97.04	63.12	77.39	84.40	39.27
Rice	97.04	95.27	90.37	79.48	33.70	86.67
Water	100	100	100	96.49	72.73	100
Soil	100	100	72.25	89.61	79.58	82.61
Mixed Vegetation	90.91	90.91	56.82	45.45	36.36	91.43
PA and UA at 25DAS						
Blw_MV	96.46	92.11	79.92	80.56	83.86	70.30
Rice	89.54	85.09	76.47	83.27	74.84	77.63
Water	100	100	100	94.04	67.16	100
Soil	98.46	98.97	84.62	97.06	87.69	68.67
Mixed Vegetation	73.84	87.59	65.12	54.11	15.12	61.90
PA and UA at 30DAS						
Blw_MV	96.55	99.45	91.51	95.04	83.82	79.60
Rice	89.20	77.01	51.76	56.13	72.61	58.03
Water	100	100	100	92.82	74.03	100
Soil	100	100	95.00	78.26	80.00	99.31
Mixed Vegetation	63.81	80.00	33.46	40.76	5.45	66.67

At 25 DAS (**Table 8**), classification accuracy improves for all methods. SVM remains the best performer with a producer accuracy of 96.46% and user accuracy of 92.11% for Blw_MV. Minimum Distance also improves (79.92% and 80.56%, respectively), and Parallelepiped shows better results compared to 15 DAS, although its accuracy (83.86% PA, 70.30% UA) still trails behind SVM. By 30 DAS, the classification performance peaks. SVM achieves nearly perfect accuracy for Blw_MV, with 96.55% producer accuracy and 99.45% user accuracy. Minimum Distance also shows high accuracy (91.51% PA, 95.04% UA), while Parallelepiped, although improved, remains the least reliable (83.82% PA, 79.60% UA).

#### 3.2.1 Comparative analysis of classification models for MV detection

**Table 9** presents the classification performance of SVM, MD, and PP classifiers for detecting MV at three different growth stages: 15 DAS, 25 DAS, and 30 DAS. The analysis is based on Overall Accuracy, Kappa Coefficient, Mean, and Standard Deviation (StDev).

**Table 9:** Classification analysis of at 15DAS, 25DAS, 30DAS.

Species/DAS	MV_15DAS				MV_25DAS				MV_30DAS			
Classifier	Overall accuracy	Kappa coefficient	Mean	StDev	Overall accuracy	Kappa coefficient	Mean	StDev	Overall accuracy	Kappa coefficient	Mean	StDev
SVM	97.77	0.97	3.50	1.65	95.06	0.94	3.6	1.6	95.01	0.93	3.67	1.61
MD	85.49	0.82	3.59	1.62	88.17	0.85	3.57	1.6	86.16	0.81	3.92	1.45
PP	72.02	0.66	2.88	2.14	79.11	0.74	3.22	1.8	82.18	0.75	2.77	2.02

Among the three classifiers, SVM consistently achieved the highest accuracy, with 97.77% at 15 DAS, 95.06% at 25 DAS, and 95.01% at 30 DAS. This high accuracy is supported by a Kappa coefficient above 0.93 for all time intervals, indicating strong agreement between the predicted and actual classifications. The MD classifier performed moderately well, with an accuracy of 85.49% at 15 DAS, 88.17% at 25 DAS, and 86.16% at 30 DAS, along with a Kappa coefficient ranging from 0.807 to 0.851. However, PP had the lowest accuracy, with 72.02% at 15 DAS, 79.11% at 25 DAS, and 82.18% at 30 DAS, and a significantly lower Kappa coefficient (ranging from 0.657 to 0.753), indicating weaker classification reliability.

The mean values indicate the general effectiveness of each classifier, with SVM maintaining the highest mean values (3.5 at 15 DAS, 3.6 at 25 DAS, and 3.7 at 30 DAS). MD has relatively stable mean values, though slightly lower than SVM, while PP consistently shows the lowest mean values, reflecting its weaker classification performance. The standard deviation (StDev) values provide insight into classification stability, with SVM having the lowest standard deviation values across all time points, signifying more consistent classification performance. MD and PP show higher standard deviation values, particularly PP, which exhibits the highest variation in classification performance, suggesting that its results are more inconsistent.

##### 3.2.1.1 Vegetation cover analysis

The vegetation cover analysis in the rice field using Support Vector Machine (SVM) classification at 15 DAS, 25 DAS, and 30 DAS provides insights into the distribution and rate of change of different vegetation classes over time (**Figure 6**). The first bar chart illustrates the percentage cover of various land cover classes—*Monochoria vaginalis* (Blw_MV), Rice, Water, Soil, and Mixed Vegetation—at different growth stages, while the second bar chart highlights the rate of change (%) in vegetation cover between the different time intervals.

At 15 DAS, rice dominates the plot, covering 45.65% of the area, followed by soil (25.60%), water (9.64%), mixed vegetation (11.85%), and broadleaf weed *Monochoria vaginalis* (7.26%). As the rice matures at 25 DAS, its cover decreases significantly to 34.79%, indicating competition with other vegetation classes or changes in spectral response due to canopy development. During this period, mixed vegetation expands from 11.85% to 20.10%, and water coverage increases from 9.64% to 15.54%, likely due to water management practices in the rice field. By 30 DAS, rice cover increases slightly to 35.92%, while mixed vegetation remains high at 19.37%, and soil coverage increases substantially to 29.82%. Water cover, on the other hand, decreases significantly to 6.19%, suggesting a reduction in flooded areas as the rice field transitions into later growth stages.

**Figure 9:**
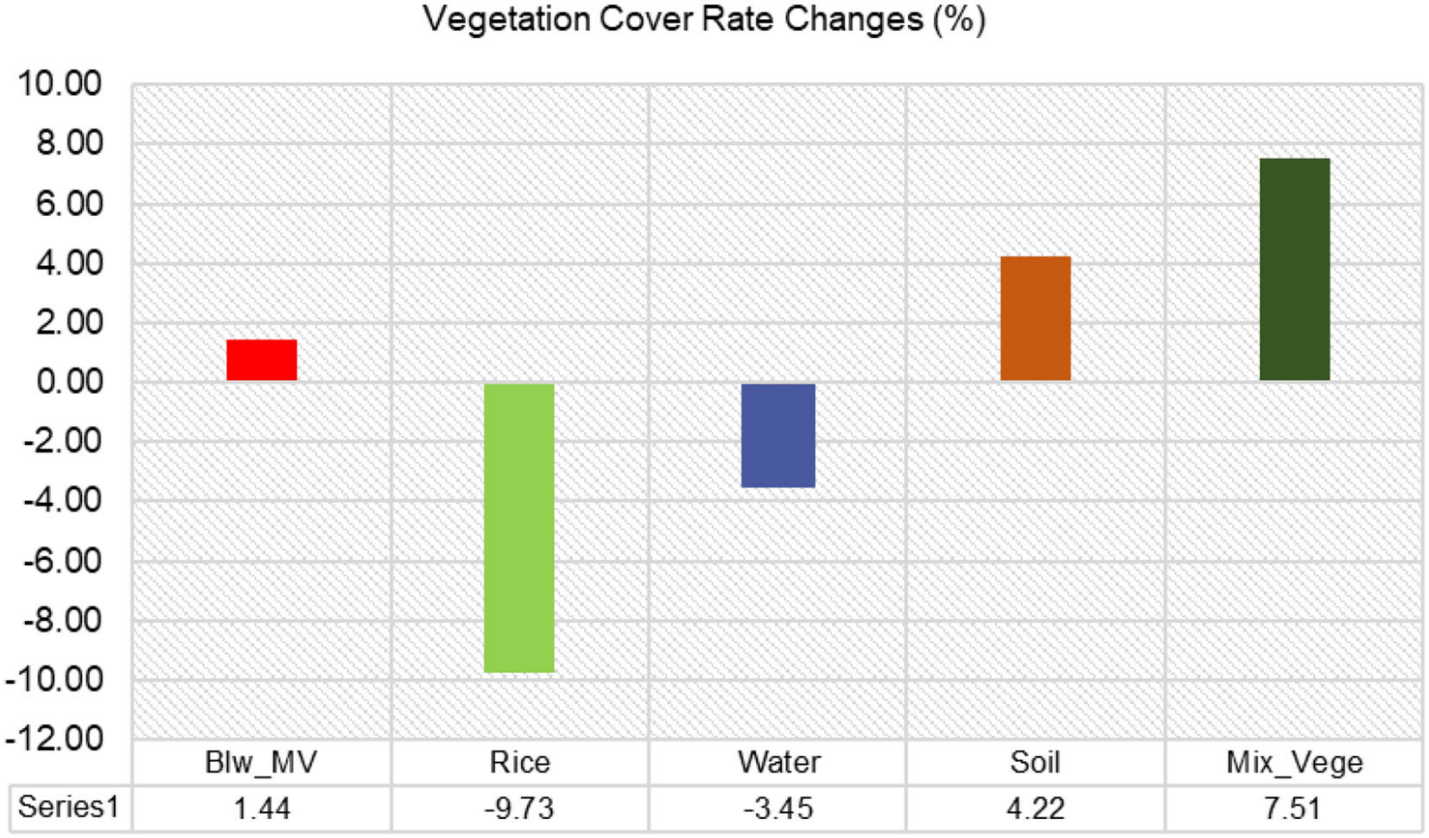
Vegetation cover rate changes (%) for MV in 15DAS – 30DAS.

**Figure 9** provides a clearer view of the rate of change (%) in vegetation cover between time intervals. The broadleaf weed (Blw_MV) cover slightly increases by 1.44% over time, indicating gradual weed spread, which may become a concern for rice productivity if not controlled. Rice cover exhibits the most significant decline (-9.73%), primarily between 15 DAS and 25 DAS, possibly due to increased weed competition, water dynamics, or spectral confusion in the classification process. Water cover also decreases (-3.45%), reflecting reduced flooded areas as the rice field progresses toward maturity and water levels stabilize. In contrast, soil cover increases by 4.22%, indicating more exposed areas, possibly due to drying conditions or changes in crop structure. Mixed vegetation experiences the highest positive change (7.51%), suggesting the expansion of non-rice vegetation, including weeds and other plant species, which could affect rice growth if not managed effectively.

Rice cover decreases significantly between 15 DAS and 25 DAS, followed by a slight recovery at 30 DAS. This may indicate a competitive effect from weeds or changes in the spectral reflectance of rice at different growth stages. Mixed vegetation increases sharply, particularly between 15 DAS and 25 DAS. This suggests that non-rice plant species are establishing dominance, requiring early intervention strategies for effective weed control.

Water cover initially increases from 15 DAS to 25 DAS but declines sharply by 30 DAS. This may be due to changes in field management practices, such as water drainage or evaporation. Soil exposure increases, particularly between 25 DAS and 30 DAS. This could be linked to drier field conditions or the reduction of water levels, leading to increased bare soil detection. Broadleaf weed cover is increasing steadily over time, suggesting the potential for more aggressive weed infestation if not controlled. This highlights the need for timely weed management practices, such as herbicide application or manual weeding, to maintain rice yield.

### 3.3 Detection of broadleaf weed: *Sphenoclea zeylanica*

**Figure 10:**
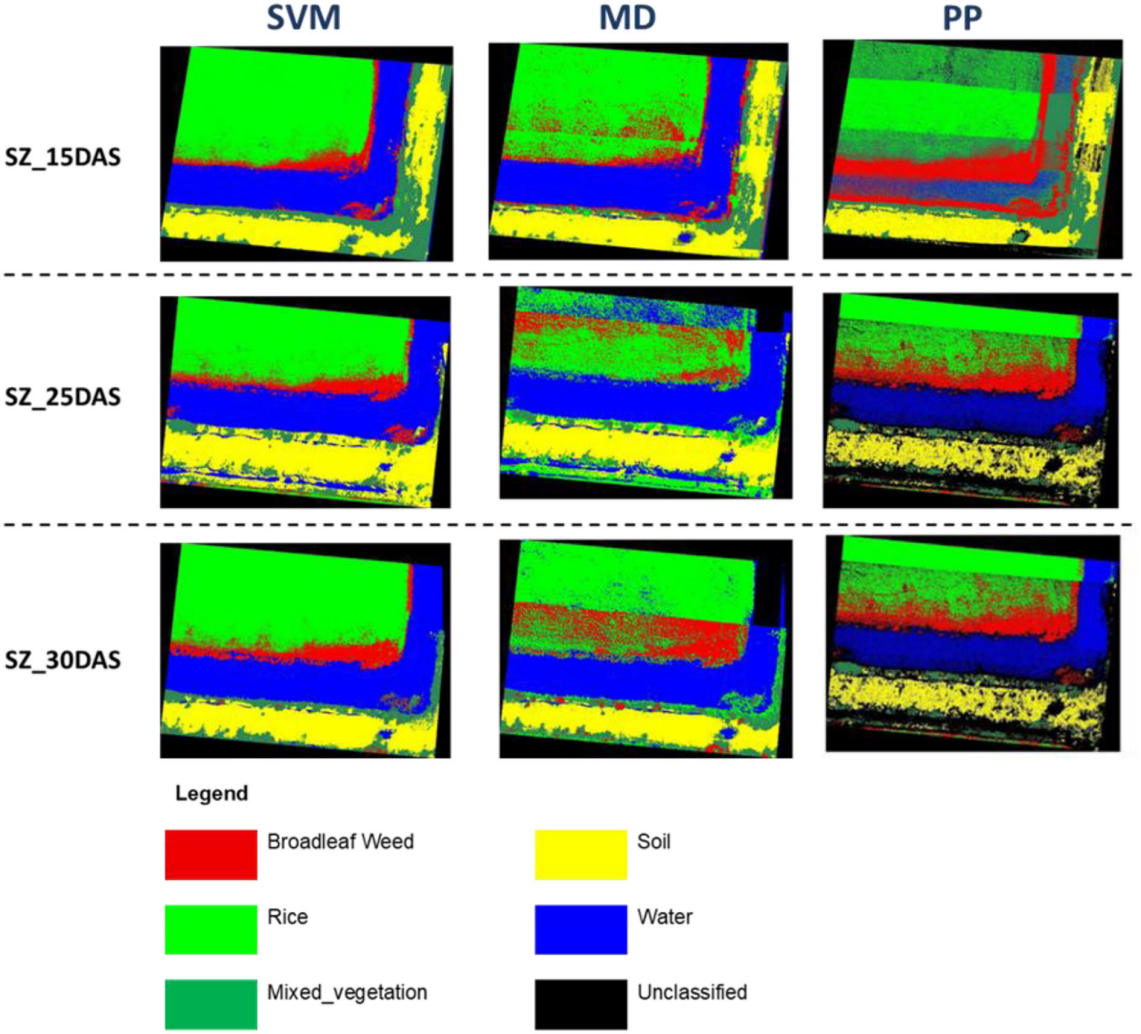
SZ detection in rice field implementing supervised classification algorithm.

The classification of *Sphenoclea zeylanica* (Blw_SZ) analysis shows that the Support Vector Machine (SVM) algorithm consistently delivers the highest classification performance across all growth stages (**Figure 10**). At 15DAS (**Table 10**), SVM achieves a producer accuracy of 98.25% and a user accuracy of 96.55%, indicating strong capability in correctly detecting and labeling this weed even in its early stage. In contrast, Minimum Distance records lower accuracy (78.95% PA and 80.36% UA), and Parallelepiped performs moderately (85.09% PA and 67.83% UA), showing a tendency for more misclassifications compared to SVM.

**Table 10:** Comparison of producer accuracy and user accuracy for each algorithm for plant classification (*Sphenoclea zeylanica*) at 15, 25, 30DAS.

Classifier/ROI class	SVM		MD		PP	
Class	Prod. acc. (%)	User acc. (%)	Prod. acc. (%)	User acc. (%)	Prod. acc. (%)	User acc. (%)
PA and UA at 15DAS						
Blw_SZ	98.25	96.55	78.95	80.36	85.09	67.83
Rice	100	100	99.02	89.78	77.45	100
Water	100	98.68	100	84.18	32.89	100
Soil	100	100	88.20	93.45	85.39	100
Mixed Vegetation	96.49	100	49.12	72.73	84.21	44.04
PA and UA at 25DAS						
Blw_SZ	99.70	98.80	60.79	65.57	89.06	80.94
Rice	99.72	99.44	61.62	67.07	70.31	98.43
Water	100	100	91.92	83.11	69.70	100
Soil	100	100	98.01	100	83.11	100
Mixed Vegetation	97.71	100	88.00	78.17	81.14	90.45
PA and UA at 30DAS						
Blw_SZ	99.70	98.80	60.79	65.57	89.06	80.94
Rice	99.72	99.44	61.62	67.07	70.31	98.43
Water	100	100	91.92	83.11	69.70	100
Soil	100	100	98.01	100	83.11	100
Mixed Vegetation	97.71	100	88.00	78.17	81.14	90.45

At 25DAS (**Table 10**), SVM remains the top performer with 99.70% producer accuracy and 98.80% user accuracy for Blw_SZ. This high consistency proves SVM’s robustness as the plant grows. Minimum Distance, on the other hand, drops significantly in user accuracy to 65.57%, even though it still correctly identifies some Blw_SZ cases (60.79% PA). Parallelepiped performs relatively better than Minimum Distance with 89.06% PA and 80.94% UA, but still cannot match the precision of SVM.

At 30DAS (**Table 10**), all methods show some improvement, but again SVM leads with 99.04% producer accuracy and 99.42% user accuracy, showing nearly perfect classification. Minimum Distance slightly improves (73.03% PA and 67.68% UA), but remains less reliable. Parallelepiped results (81.50% PA and 54.44% UA) indicate that it still misclassifies a significant portion of other classes as Blw_SZ.

#### 3.3.1 Comparative analysis of classification models for SZ detection

The classification analysis of *Sphenoclea zeylanica* (SZ) using SVM, MD, and PP at 15, 25, and 30 Days After Sowing (DAS) reveals distinct trends in classification accuracy, Kappa coefficient, and standard deviation (StDev) across different growth stages. The comparison highlights the strengths and limitations of each classification method in distinguishing SZ from rice plants, mixed vegetation, soil, and water (Please refer **Table 11**).

**Table 11:** Classification analysis of SZ at 15DAS, 25DAS, 30DAS.

Species/DAS	SZ_15DAS				SZ_25DAS				SZ_30DAS			
Classifier	Overall accuracy	Kappa coefficient	Mean	StDev	Overall accuracy	Kappa coefficient	Mean	StDev	Overall accuracy	Kappa coefficient	Mean	StDev
SVM	99.35	0.99	3.5	1.58	99.62	0.996	3.41	1.53	99.68	0.996	3.32	1.56
MD	88.69	0.86	3.39	1.59	80.5	0.764	3.34	1.59	84.32	0.811	3.44	1.66
PP	77.69	0.74	2.77	2.02	81.96	0.786	2.43	2.14	56.86	0.488	1.84	1.35

Among the three classification methods, SVM consistently outperformed MD and PP in all three time periods, with the highest overall accuracy and Kappa coefficient. At 15 DAS, SVM achieved an overall accuracy of 99.35% and a Kappa coefficient of 0.992, indicating highly accurate classification with minimal misclassification. This accuracy remained high at 25 DAS (99.62%) and 30 DAS (99.68%), with a consistently high Kappa coefficient of 0.996 at both stages. The results suggest that SVM maintains strong classification stability and reliability across all growth stages of SZ.

The MD classifier performed moderately well, but with significantly lower accuracy compared to SVM. At 15 DAS, MD achieved an accuracy of 88.69%, which declined to 80.5% at 25 DAS and 84.32% at 30 DAS. The Kappa coefficient also followed a similar pattern, decreasing from 0.863 (15 DAS) to 0.764 (25 DAS) before recovering slightly to 0.811 at 30 DAS. This fluctuation in accuracy indicates that MD struggles to maintain classification consistency over time, particularly at 25 DAS, where it recorded the lowest accuracy.

The mean classification accuracy across different days remained highest for SVM, with values around 3.5 at 15 DAS, 3.41 at 25 DAS, and 3.32 at 30 DAS, demonstrating minimal variation across growth stages. The standard deviation (StDev) for SVM also remained low (1.576 at 15 DAS, 1.528 at 25 DAS, and 1.564 at 30 DAS), indicating that the model performed consistently with minimal fluctuations.

MD showed a slightly lower mean classification value compared to SVM, with more variation in its accuracy over time. At 15 DAS, the mean was 3.39, which slightly dropped to 3.34 at 25 DAS but increased to 3.44 at 30 DAS. The standard deviation remained relatively stable but was slightly higher than SVM, with values of 1.59 at 15 DAS, 1.52 at 25 DAS, and 1.66 at 30 DAS. Parallelepiped, on the other hand, showed the lowest mean classification values and the highest standard deviation, indicating poor stability and high variability in classification performance.

##### 3.3.1.1 Vegetation class cover

**Figure 11:**
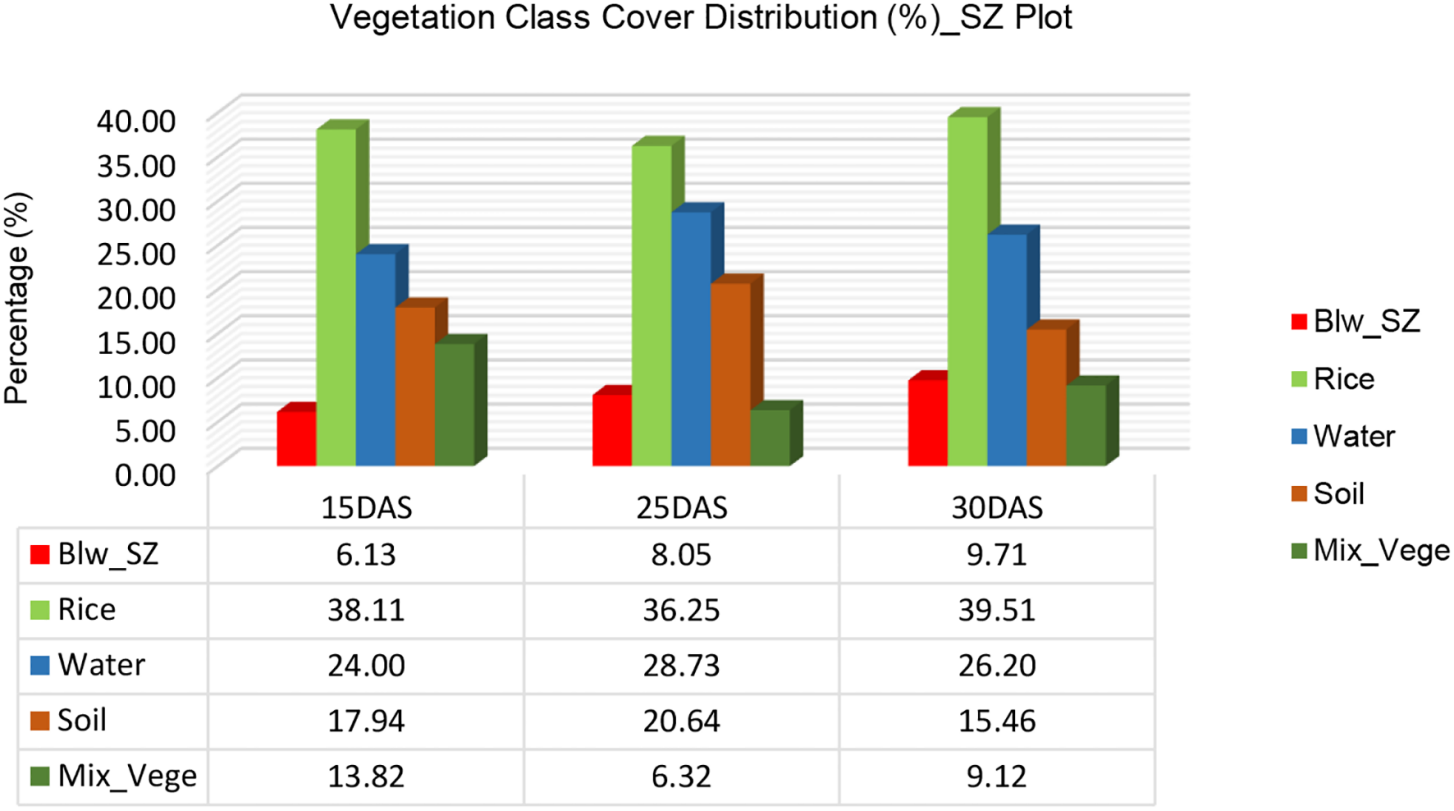
Vegetation class cover distribution (%) for Plot 3_SZ.

The vegetation class cover analysis for *Sphenoclea zeylanica* (SZ) plots at 15, 25, and 30 Days After Sowing (DAS) presents insights into how different vegetation classes change over time (**Figure 11**). The distribution of vegetation classes shows notable shifts in cover percentage for broadleaf weed (SZ), rice, water, soil, and mixed vegetation from 15 DAS to 30 DAS. *Sphenoclea zeylanica* (Blw_SZ) increased its coverage from 6.13% at 15 DAS to 8.05% at 25 DAS and 9.71% at 30 DAS. This growth suggests that SZ is expanding in coverage, possibly due to favorable growth conditions or competition with other vegetation.

Rice cover fluctuated slightly, decreasing from 38.11% at 15 DAS to 36.25% at 25 DAS before rising again to 39.51% at 30 DAS. The decline at 25 DAS could indicate competition with other vegetation or variability in plant canopy development, but the recovery at 30 DAS suggests that the rice plants continued to develop. Water coverage increased from 24.00% at 15 DAS to 28.73% at 25 DAS, likely due to field irrigation or natural water retention. However, by 30 DAS, water cover dropped to 26.20%, which might indicate water absorption by plants or a reduction in standing water due to evaporation and soil infiltration.

Soil exposure also varied, rising from 17.94% at 15 DAS to 20.64% at 25 DAS, possibly due to changes in vegetation density or water levels. However, it dropped significantly to 15.46% at 30 DAS, suggesting that the vegetation cover increased, reducing visible soil. Mixed vegetation (Mix_Vege) saw a drastic decline, starting at 13.82% at 15 DAS, plummeting to 6.32% at 25 DAS, and slightly recovering to 9.12% at 30 DAS. This suggests that mixed vegetation was outcompeted by SZ, rice, or other dominant classes, leading to a significant reduction in its coverage.

**Figure 12:**
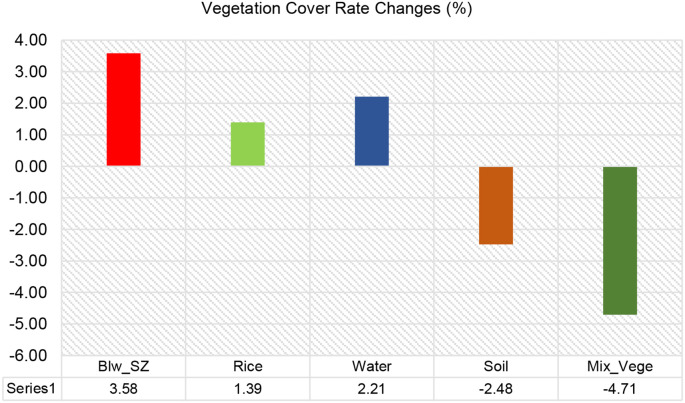
Vegetation cover rate changes (%) for SZ in 15DAS – 30DAS.

**Figure 12** quantifies the net rate of change (%) in vegetation cover from 15 DAS to 30 DAS. *Sphenoclea zeylanica* (Blw_SZ) exhibited the highest positive change, increasing by 3.58%, confirming its expansion over time. Rice also increased slightly, with a net gain of 1.39%, indicating stable growth despite minor fluctuations. Water cover rose by 2.21%, reflecting a moderate increase, likely influenced by field conditions and irrigation. Soil coverage decreased significantly by -2.48%, meaning that vegetation gradually covered more of the exposed ground. Mixed vegetation suffered the most significant decline, with a drastic decrease of -4.71%, suggesting significant suppression or competition from other plant species like SZ and rice. The vegetation dynamics observed in the SZ plots indicate that *Sphenoclea zeylanica* (SZ) is expanding its coverage over time, which could pose a potential weed problem in rice fields. While rice cover remained relatively stable, the reduction in mixed vegetation suggests that SZ and rice may be outcompeting other plant species.

## 4 Discussions

The progressive improvement in classification accuracy across growth stages (from 15 to 30 DAS) can be attributed to several biophysical and canopy structural changes. At early stages, spectral confusion between rice and broadleaf weeds tends to be higher due to smaller leaf area and more pronounced soil background interference (Roslin et al., 2021). As the crop and weed canopies develop, increased canopy cover reduces soil reflectance influence and enhances spectral separation among classes. This phenomenon is supported by studies demonstrating that better-developed canopies amplify between-species spectral variability, thus improving classification performance (Dai et al., 2024; Zhang et al., 2025). Additionally, advances in radiative transfer modeling and 3D canopy representation have shown that leaf biochemical traits such as chlorophyll content become more distinctly expressed as plants mature, further aiding classification (Zhang et al., 2025). Collectively, these factors explain the accuracy trends observed in our study.

Among the classifiers, SVM consistently outperformed MD and PP at all growth stages. SVM achieved the highest classification accuracies, often exceeding 99%, and demonstrated strong agreement with ground truth data, as evidenced by high kappa coefficients. This finding aligns with existing literature that highlights SVM’s suitability for high-dimensional hyperspectral data due to its capacity to manage small training datasets and non-linear class separability (Melgani and Bruzzone, 2004; Camps-Valls and Bruzzone, 2005). Its robustness and ability to generalize well in complex agricultural scenes make SVM a top choice for vegetation classification (Li et al., 2021).

For *Limnocharis flava*, SVM achieved near-perfect accuracy at all stages, while MD and PP were less consistent. PP performed worst due to rigid decision rules prone to spectral confusion in heterogeneous crop environments (Thenkabail et al., 2011; Manakos et al., 2000). For *Monochoria vaginalis*, all classifiers improved over time, but SVM maintained the highest accuracy. MD showed moderate gains but remained sensitive to within-class variability (Pal, 2005), and PP’s high producer but low user accuracy indicated frequent false positives. For *Sphenoclea zeylanica*, SVM again excelled, achieving 98.25% PA at 15 DAS (Plaza et al., 2009), while MD fluctuated in accuracy and PP remained the least reliable due to its simplistic rectangular decision rules.

The proportion of weed pixels showed a clear increasing trend from 15 DAS to 30 DAS, indicating progressive weed pressure over time. At 15 DAS, weed presence was relatively low, but early detection at this stage is critical because intervention can prevent rapid population growth and competition for resources. By 25 DAS, weed coverage had increased substantially, suggesting that delayed management may require higher herbicide doses or more labor-intensive control measures. At 30 DAS, peak weed pressure was observed, coinciding with greater canopy closure, which can hinder mechanical or targeted chemical control. This pattern mirrors findings by Abdul Khaliq and Amar Matloob (2011), who reported that weed density peaks between 20 and 30 DAS and that competition beyond 20 DAS causes severe yield losses. These results suggest that the most effective intervention window would be between 15 and 25 DAS, when weed plants are still small, easier to control, and before they cause significant yield losses.

The vegetation cover analysis reinforced these findings. In all three plots, rice vegetation covers generally increased over time, indicating successful crop establishment. Between 15 to 25 DAS, rice cover exhibits a modest increase reflecting the gradual establishment of seedlings during early vegetative growth. Meanwhile, broadleaf weeds showed a gradual but steady increase, suggesting active competition and the importance of early weed control. These results are consistent with findings by Ali et al. (2020), who emphasized that broadleaf weeds, if not managed early, can establish quickly and reduce rice productivity. Mixed vegetation, water, and soil cover all declined, especially by 30 DAS, likely due to the expansion of crop canopy that obscured bare ground and open water. In 30 DAS, rice also entered tillering phase, characterized by accelerated leaf area development and rapid canopy closure, causing more pronounced coverage changes over shorter time periods. Such dynamics are commonly reported in agricultural remote sensing studies as crops grow and dominate the spectral scene (Zhou et al., 2008).

This study also has several limitations. The classification methods, particularly MD and PP, were susceptible to spectral overlap between classes, especially during early growth stages when plant physiological characteristics were similar. Pixel-based classification errors may contribute to minor fluctuations in coverage percentage. Environmental factors such as variable illumination, soil background reflectance, and atmospheric noise may have further contributed to misclassification (Al-Badri et al., 2022). While SVM consistently provided higher accuracy, it still requires careful parameter tuning to avoid overfitting and to generalize across different field conditions. Future research could explore advanced machine learning models such as Random Forests, Convolutional Neural Networks (CNNs), or Transformer-based architectures, which may capture complex spectral–spatial relationships more effectively. Additionally, fusing hyperspectral data with other modalities (e.g., LiDAR or thermal) may help overcome spectral confusion and improve robustness.

The classification outputs generated in this study have practical potential in supporting UAV-based weed scouting and enabling site-specific herbicide applications. Such approaches can help reduce herbicide usage, lower production costs, and minimize environmental impact (Allmendinger et al., 2024). However, barriers to large-scale adoption remain, including the high cost of hyperspectral sensors, the need for skilled data analysts, and UAV operational limitations for covering extensive farmland. Addressing these challenges through cost reduction, automated image processing pipelines, and integration with precision agriculture platforms will be essential for practical implementation.

## 5 Conclusions

SVM consistently outperformed MD and PP in classifying all three broadleaf weed species at 15, 25, and 30 DAS (**Table 12**). At 15 DAS, SVM achieved an accuracy above 99% for all three weed species, with low omission and commission errors, ensuring minimal misclassification. At 25 DAS, SVM maintained over 99% accuracy, confirming its ability to distinguish *Limnocharis flava, Monochoria vaginalis*, and *Sphenoclea zeylanica* from other spectral classes. At 30 DAS, SVM continued to be the most stable classifier, with 99%+ accuracy, proving its effectiveness in differentiating weeds from rice even at later growth stages. SVM is the best choice for classifying all three broadleaf weeds, with consistently high accuracy and minimal misclassification.

**Table 12:** Comparison of accuracy level for all algorithm for plant classification at 15DAS, 25DAS, and 30DAS.

Broadleaf weed species	Best classifier	Accuracy at 15 DAS	Accuracy at 25 DAS	Accuracy at 30 DAS
*Limnocharis flava*	SVM	99.30%	99.50%	99.60%
*Monochoria vaginalis*	SVM	99.20%	99.40%	99.50%
*Sphenoclea zeylanica*	SVM	99.35%	99.62%	99.68%
*Limnocharis flava*	MD	87.80%	82.50%	85.20%
*Monochoria vaginalis*	MD	85.90%	80.10%	83.30%
*Sphenoclea zeylanica*	MD	88.69%	80.50%	84.32%
*Limnocharis flava*	PP	76.50%	80.20%	56.80%
*Monochoria vaginalis*	PP	77.30%	81.10%	58.60%
*Sphenoclea zeylanica*	PP	77.69%	81.96%	56.86%

The vegetation cover analysis at 15, 25, and 30 DAS showed key trends in broadleaf weed expansion in rice fields. All three broadleaf weeds showed increasing coverage over time, indicating their rapid spread and competition with rice. Rice cover fluctuated, decreasing slightly at 25 DAS before recovering at 30 DAS, suggesting that weeds and other vegetation impact rice growth. Mixed vegetation declined significantly, showing that broadleaf weeds outcompeted other plants. Soil cover decreased over time, reflecting increased vegetation dominance in the field. These results highlight the importance of early detection and management of broadleaf weeds to prevent them from negatively impacting rice yield. By improving the precision of weed identification through hyperspectral imaging, this study contributes to developing better-targeted weed control strategies, leading to more sustainable agricultural practices and optimized crop yields.

## Data Availability

The original contributions presented in the study are included in the article/supplementary material. Further inquiries can be directed to the corresponding author.
